# The Effect of Heat on Dentine

**Published:** 1898-10

**Authors:** G. W. Cook

**Affiliations:** Chicago, Illinois


### The Effect of Heat on Dentine.

BY G. W. COOK, D.D.S., CHICAGO, ILLINOIS.

Abstract of Paper read before the Tri-State Dental Meeting, Put-in-Bay, Ohio, June, 1898.

In investigating as to how much heat could be used in a tooth, I found three factors to be taken into consideration, viz: the effect of heat on bacteria, the amount of moisture extracted and the effect on dentine. The first of these is of the greatest importance, a temperature only 6° above the body temperature will render micro-organisms harmless; 90° C. in most all cases kill bacteria in pulp canals, by an application of ten minutes when a root canal drier is used. But after a number of experiments at various degrees of heat, I found it required about 130° C. of three applications of four and a half minutes each to thoroughly sterilize root canals. A course of treatment of this kind necessarily causes the loss of moisture, and what the effect of such a loss on tooth structure will be is a question.

Of course, all living tissues contain more or less water. But water, as water, does not enter into the chemical constitution of the molecules, but is simply held by inhibition, *i. e.*, the power of all colloid substances to imbibe (take up) a certain amount of water, which causes them to swell up and keep their shape as well as their toughness and flexibility (not easily broken). It might truly be stated that the function of water in all bone tissue, in so far as its resistance is concerned, is to keep the colloid material in an elastic, tough condition, as opposed to the brittle, non-elastic condition of dry, fibrous tissue. So it is readily seen that, while water is not an element in tooth structure at all, its presence is necessary for the normal elasticity and toughness of the tooth tissue.

On the other hand, if the water is driven off by heat or otherwise, a shrinking follows, and instead of being elastic and flexible, the teeth become brittle and inelastic. From these facts the brittleness of old, dry teeth, or teeth which have been subjected to a degree of heat to drive off water, can be explained. Although it is hard to make out any contraction in the dentine or separation of it from the enamel, yet it is a well proven fact that after drying, the enamel is easily broken off from the dentine and the whole tooth structure rendered more brittle. This can only be explained by assuming that the water held mechanically by the colloid matrix of the tooth has been driven off, thus rendering the organic structure brittle and non-elastic.

From the above, the function of the water as regulating the integrity of the tooth is evident as well as the effect of drawing off the water upon the elasticity and flexibility of the tooth.

I found that the application of a temperature of 130° C. by means of the root drier was sufficient to destroy bacteria in pulp canals in three applications of four and a half minutes each. Such treatment generally reduced the weight of the tooth from 0.65 to 1 percent, the loss being due to the driving off of water. While such an application of heat aids very materially in the destruction of bacteria, and in rendering the pulp canal aseptid, the question concerning its action on the resisting power of the tooth structure must be taken into consideration. As already stated, such a degree of heat has no chemical effect whatever on the inorganic salts which enter into the earthy or hard structure of the tooth. Also it might be stated in experimenting on the resisting power of teeth towards pressure, both before and after the use of heat in the manner already described, I should detect no difference in resistance; and, further, I will say that the results of my experiments in this line correspond very closely with those of Black. These experiments indicate that the resistance of the tooth structure is unimpaired by the use of the root drier.

After one has removed by mechanical means all the *debris* from the pulp canal, the application of the root drier at a temperature of about 90° to 150° C. for a very few minutes would not be out of place. But if you raise the temperature to where you get the hissing sound with root drier, which is 170° C. there will be a drying out of the organic substance, thus rendering the teeth more brittle.

